# Structural and ensemble-based mechanistic insights into cytoplasmic dynein-1

**DOI:** 10.3389/fmolb.2026.1832580

**Published:** 2026-05-08

**Authors:** Amit Kumawat

**Affiliations:** Department of Physics, University of Cagliari, Cagliari, Italy

**Keywords:** allostery, dynein-1, integrative modelling, MD simulations, motor proteins

## Abstract

Cytoplasmic dynein-1 is a multi-subunit motor that transports diverse cargoes toward microtubule minus-ends, playing crucial roles in intracellular organisation and cell division. Over the past decade, X-ray crystallography and cryo-EM approaches have captured numerous high-resolution structures of dynein’s components and complexes ranging from motor domain structures and microtubule binding domain (MTBD)-tubulin complexes, to the autoinhibited and open dynein dimers, activated dynein-dynactin-adaptor assemblies, and LIS1-bound intermediates. These findings have highlighted dynein’s architecture and partial conformational cycle, including key intermediates in its activation and assembly with dynactin and cargo adaptors. However, a unified molecular model that links ATP hydrolysis at multiple AAA+ sites to the coordinated motions of dynein’s components and their regulation by dynactin, cargo adaptors, and LIS1 remains elusive. Existing mechanistic models rely on discrete structural states and simplified kinetic schemes, which fail to capture conformational heterogeneity and regulator-specific effects. In this mini review, we summarise the current structural and mechanistic knowledge of cytoplasmic dynein-1, highlighting the motor’s stepping mechanism and cofactor functions. We then discuss computational approaches including molecular dynamics simulations, enhanced sampling techniques, and integrative modelling that can merge these specific states into quantitative conformational landscapes. This ensemble view will be helpful for understanding dynein’s mechanism from a dynamical perspective.

## 1 Introduction

Cytoplasmic dynein-1 (dynein) is the primary minus-end-directed microtubule motor in animals and fungi, responsible for transporting organelles, vesicles, RNAs, protein complexes, and even viruses across vast intracellular distances (Kardon and Vale, 2009; Dodding and Way, 2011; Roberts et al., 2013; Reck-Peterson et al., 2018). It is also involved in positioning the spindle and segregating chromosomes during mitosis (Gassmann, 2023). Loss of function mutations and defects in dynein or its regulators cause neurodegenerative diseases and are lethal at the organismal level in mammals (Hafezparast et al., 2003; Eschbach and Dupuis, 2011; Hoang et al., 2017; Di Pizio et al., 2022; Park et al., 2024). Unlike different kinesin motors, the cell relies on this single cytoplasmic dynein isoform for most long-range minus-end-directed transport in the cytoplasm and on an array of cofactors to regulate its activity (Cianfrocco et al., 2015). Structurally, dynein is a ∼1.4 MDa dimer of two identical heavy chains, each of which associates with multiple accessory subunits (intermediate, light-intermediate, and light chains) (Canty et al., 2021). The heavy chain contains an N-terminal tail domain (for dimerization and cargo/adaptor/dynactin binding) and a C-terminal motor domain. The motor domain is formed of six AAA+ ATPase modules (AAA1-AAA6) arranged in a ring with a protruding coiled-coil stalk supported by the buttress that terminates in a microtubule binding domain (MTBD) (Schmidt and Carter, 2016). An elastic linker emerges from the ring and connects to the tail, and the linker undergoes large conformational swings relative to the ring that generate force and displacement (Roberts et al., 2009). The motor domain is characterised by ATP binding at four of the six sites, but only three (AAA1, AAA3, AAA4) hydrolyse it, while AAA5 and AAA6 are non-hydrolytic. AAA1 is the principal ATPase driving the motor, whereas AAA3 and AAA4 serve regulatory roles through allosteric coupling (DeWitt et al., 2015; Liu et al., 2020).

Despite dynein’s large size and complexity, X-ray crystallography and cryo-EM studies in the 2010s resolved structures of dynein motor domains from yeast and *Dictyostelium*, exhibiting the arrangement of the AAA+ ring, linker, stalk, and buttress elements (Carter et al., 2011; Kon et al., 2012; Schmidt et al., 2012; 2015). Subsequent cryo-EM investigation provided snapshots of dynein’s interactions with microtubules and autoinhibited phi conformation formed due to self-dimerization of its motor domains in the absence of cargo-adaptors or dynactin (Torisawa et al., 2014). Regulators such as LIS1 and Nde1 participate in relieving the phi state and promoting assembly of the dynein-dynactin-adaptor (DDA) complex (Kusakci et al., 2024; Singh et al., 2024; Yang et al., 2026). Furthermore, the DDA complex can move with high processivity and force, especially if two dynein dimers are recruited per dynactin (Chaaban and Carter, 2022). More recently, cryo-EM analysis of full length human dynein across multiple nucleotide and microtubule-bound states has organised these snapshots into a mechanochemical framework and revealed additional intermediate conformations (Chai et al., 2025). All these findings have provided a state-specific molecular level information about dynein. However, existing models that depict dynein’s cycle as a series of discrete states fail to capture the conformational flexibility and the stochastic timing of events. Hence, several questions regarding the net directional motions of dynein motors and how events at distal regions of multiple subunits are allosterically linked remain only partially answered.

In this mini review, we first provide an overview of the structural states of cytoplasmic dynein-1 and what they have revealed about its mechanism. We then discuss open questions highlighting the coordination of AAA+ ATPase subunits, dynein’s stepping mechanism, and its regulation by dynactin/adaptors/LIS1. Finally, we describe emerging ensemble-based approaches that are beginning to bridge the gaps between static structures and dynamic function. These multidisciplinary approaches, ranging from molecular dynamics (MD) simulations to cryo-EM heterogeneity analysis and integrative modelling, offer a promising path to map out dynein’s full energy landscape and understand how specific regulators modulate that landscape.

## 2 Mechanistic insights into the dynein motor using experimental structures

Over the past decade, X-ray crystallography, cryo-EM, and *in situ* cryo-ET have captured several conformations of dynein and its regulators including isolated nucleotide-bound motor domains, the autoinhibited (phi) dynein, open dynein, DDA complexes, and LIS1 stabilised intermediates (Cho and Vale, 2012). Early efforts in the 2010s focused on obtaining high resolution structures of the isolated dynein’s motor domains in different nucleotide states (ATP analogs, ADP, apo), providing insight into its mechanochemical cycle, namely, priming and the powerstroke, that couple the ring to the linker and stalk. For example, crystal structures and cryo-EM reconstructions showed the linker in a bent conformation with ATP or analog-bound states and docked (straight) onto the ring in ADP bound or inorganic phosphate (Pi) released states (Carter et al., 2011; Kon et al., 2012; Schmidt et al., 2012; Roberts et al., 2012; Bhabha et al., 2014). Subsequent cryo-EM studies of dynein motor components bound to microtubules captured the linker’s conformational swing from a primed (pre-stroke) to a straight (post-stroke) position, generating force (Redwine et al., 2012; Lin et al., 2014; Schmidt et al., 2015). Simultaneously, the stalk coiled coil undergoes a register shift (between α and β registry) that switches the MTBD between low and high affinity states (Gibbons et al., 2005; Carter et al., 2008; Choi et al., 2011; Nishikawa et al., 2016; Lacey et al., 2019). The buttress helix (from AAA5) interacting with the stalk undergoes conformational change as AAA1’s nucleotide pocket opens, suggesting it transmits allosteric signals from the ring to the stalk (Schmidt et al., 2012; 2015). Zhang and coworkers suggest that this allosteric communication is bidirectional, with microtubule binding feeding back through the stalk-buttress axis to stabilise the α registry, promote wider AAA1 opening, and accelerate ADP release (Chai et al., 2025). Recent structural and biophysical studies indicate that these changes in stalk registry, buttress conformation, AAA1 pocket opening, and linker position can occur in at least two partially decoupled phases, rather than as a perfectly synchronised switch (Cleary et al., 2014; Chai et al., 2025). Moreover, mutations that block ATP hydrolysis at AAA3 reduce AAA1’s hydrolytic activity, thus allosterically modulating AAA1’s kinetics and indirectly the timing and amplitude of the stroke (Bhabha et al., 2014).

At the level of the intact dynein complex, cryo-EM structures revealed the phi autoinhibited conformation, in which two motor domains are packed in a cross legged arrangement, with the linker of one motor contacting the AAA ring of the other, constraining the motors in low microtubule affinity geometries and limiting their ability to engage microtubules (Zhang et al., 2017). The phi state locks both motors in a low microtubule affinity state and its release is thus a prerequisite for motility (Torisawa et al., 2014). Mutation studies and recent cryo-EM structures of dynein bound to LIS1 provide insight into the mechanism of phi dynein changing into an open conformation (Gillies et al., 2022; Karasmanis et al., 2023). Open dynein shows higher microtubule association but still moves poorly in the absence of dynactin, suggesting that the heads must be correctly oriented relative to one another and the microtubule lattice for productive stepping (Can et al., 2019; Rao et al., 2024). Thus, as an additional activation step, cryo-EM structures of DDA complexes with adaptors such as JIP3, BicD2 and BICDR1 showed that dynactin arranges dynein’s two heads in an open parallel configuration on the same side of the dynactin Arp1 filament (McKenney et al., 2014; Schlager et al., 2014; Urnavicius et al., 2015; Singh et al., 2024). Two dynein motors can bind to adjacent or near-adjacent tubulin subunits, revealing an asymmetry between the two heads and suggesting a division of labour during stepping (Chaaban and Carter, 2022). Furthermore, some adaptors (like BICDR1 and HOOK3) recruit two dynein dimers to one dynactin, effectively increasing force output and faster motion, illustrating that different cargo adaptors can tune dynein’s motile properties by changing the stoichiometry and geometry of the complex (Urnavicius et al., 2018; Elshenawy et al., 2019; Aslan et al., 2025).

**FIGURE 1:**
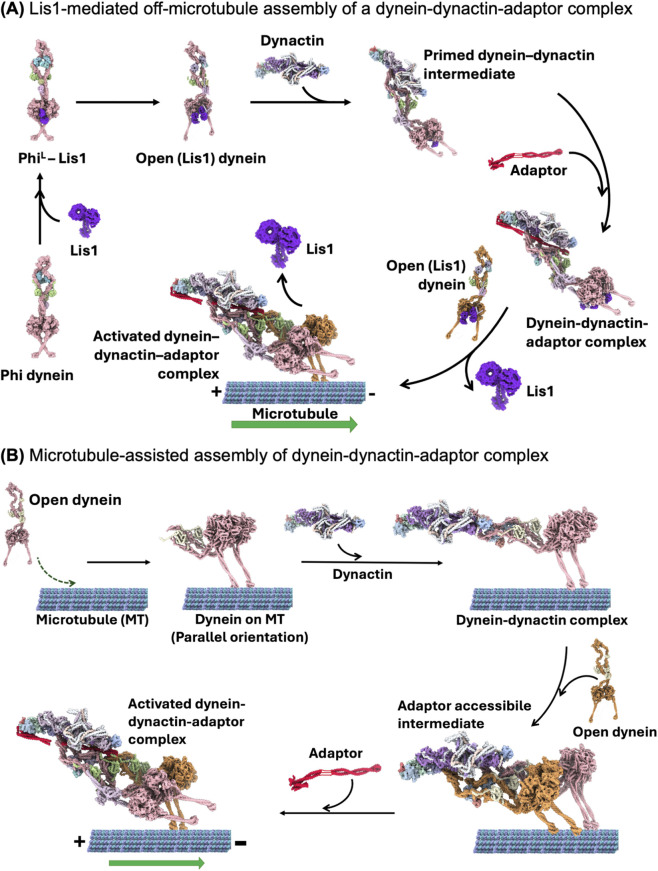
Two proposed pathways for assembly of activated dynein-dynactin-adaptor complexes. Structural representations are schematic and are assembled from representative experimental structures; unresolved regions and subunits, particularly within dynactin and associated factors, are omitted because they are absent in the available models. **(A)** In the LIS1-mediated off-microtubule pathway, autoinhibited phi dynein (PDB: 9E12) is converted into a LIS1-bound opening intermediate (PDB: 9E13), followed by an open dynein-LIS1 state that is competent for assembly with dynactin. This generates a primed dynein-dynactin intermediate, which is stabilised by binding of an activating cargo adaptor to form a dynein-dynactin-adaptor (DDA) complex. LIS1 dissociates from most complexes at or near movement initiation, and the activated complex subsequently engages the microtubule for minus-end-directed transport. **(B)** In the microtubule-assisted pathway, dynein first binds the microtubule lattice, where the motor domains adopt a more parallel/aligned orientation (PDB: 9DGQ). This MT-bound dynein can then recruit dynactin in the absence of adaptor, forming an adaptor-free dynein-dynactin intermediate on the microtubule (PDB: 9DGR). Subsequent adaptor binding converts this state into an activated DDA complex capable of processive minus-end-directed motility.

Recent studies have been focused on determining the transition states mediated by LIS1, a dynein regulator containing two WD40 β propeller domains and its cofactor Nde1/NdeL1 (Gillies et al., 2022; Reimer et al., 2023; Chai et al., 2025; Yang et al., 2026). Structural findings show that in yeast motor domain constructs, LIS1 stabilises an intermediate state (chi conformation) in which two LIS1 dimers wedge between the motor domains, contacting AAA3/AAA4 (ring site) and the stalk (or AAA5/AAA6) of the opposing motor (Karasmanis et al., 2023). Rather than completely blocking the powerstroke, LIS1 binding in these states prolongs force resisting conformations and slows force induced detachment, especially under load (Toropova et al., 2014; Kusakci et al., 2024). Another role of LIS1 is to facilitate dynein’s recruitment to dynactin-adaptor complexes (Elshenawy et al., 2020; Singh et al., 2024). In full length human dynein, a single LIS1 dimer binds asymmetrically to one side of the phi dimer (Figure 1A), inducing a modest rotation between the heads and a twist of the neck region that lowers the barrier to opening. Nde1 accelerates formation of this phi-like (PhiL)-LIS1 intermediate (Nguyen et al., 2025; Yang et al., 2026). LIS1 can also interact with dynactin’s p150 arm and dynein intermediate chain elements, although the precise geometry may vary between systems (Okada et al., 2023; Kendrick et al., 2025). These findings support an off-microtubule, LIS1-mediated assembly pathway toward a dynactin-competent dynein state, as summarised in Figure 1A. Furthermore, a nucleotide code across AAA1/AAA3/AAA4 coordinates LIS1 binding affinity and stoichiometry and can determine whether LIS1 binding promotes opening versus phi-like states (closed conformations) (DeSantis et al., 2017; Geohring et al., 2026). In contrast, a recent preprint proposes an alternative, microtubule-assisted route for assembly of the DDA complex (Figure 1B), in which dynein first binds microtubules, aligns its motors, and forms an adaptor-independent dynein-dynactin complex that subsequently recruits adaptors on the lattice (Rao et al., 2024). This view complements the classical off-microtubule LIS1-mediated assembly model and suggests that multiple assembly pathways may coexist in cells.

In summary, structural studies have provided a detailed view of dynein’s conformations and interactions suggesting (a) how dynein’s motor domains change with nucleotide state (linker bending, ring closure/opening, stalk twisting), (b) how the two motors can be packed together or separated (phi vs. open), (c) how dynactin and adaptors engage dynein’s tail and align the motors for processivity, and (d) how LIS1 binding can stabilise a key intermediate in the activation pathway.

## 3 Allosteric coordination and open mechanistic questions

Experimental structures suggest that dynein’s mechanochemical cycle is governed by an interconnected allosteric network instead of a linear sequence of states (Niekamp et al., 2019; Canty and Yildiz, 2020). Within a single motor domain, ATP binding and hydrolysis at AAA1 is regulated by the nucleotide state of AAA3, by contacts between the linker and AAA2, and by the stalk-buttress ensemble that couples the ring to the MTBD (Kon et al., 2009; Niekamp et al., 2019). Structural studies show that ATP at AAA1 can in principle bend the linker, but this conformational change is effectively blocked or repressed when AAA3 is also bound with ATP or transition state mimics, disfavouring the powerstroke and creating a repressed checkpoint state (Bhabha et al., 2016). At the same time, the stalk coiled coil and buttress must propagate ring rearrangements over ∼15–20 nm to tune MTBD affinity, while also communicating information in the reverse direction when the MTBD engages or releases the microtubule (Uchimura et al., 2015; Nishida et al., 2020). How these motions are partitioned into local, fast fluctuations versus rare, large-scale transitions and how these events are translated into the kinetics and energetics of nucleotide binding/release and MTBD’s affinity towards microtubule remains poorly understood. The conformational ensembles resolved so far suggest that linker bending, AAA1 pocket relaxation, and stalk registry switching are only loosely coupled, with, for example, closed AAA1 pockets observed together with straight but undocked linkers or intermediate stalk registries, raising the question of how often motors visit partially decoupled states.

Another set of mechanistic coordination emerges at the level of the dynein dimer and its regulators. Single molecule and structural studies together indicate that the two heads do not follow a strictly deterministic stepping pattern, instead, they share load in a probabilistic manner (Qiu et al., 2012; DeWitt et al., 2012; Lippert et al., 2017). This implies interhead communication that is likely mediated through the flexible tail and the accessory chains, but the structural basis of how forces reshape the AAA rings, stalk-buttress geometry, or linker docking in each head is still unclear (Gennerich et al., 2007; Walter et al., 2012). Autoinhibited phi dimers, their LIS1-bound intermediate states, open dynein, and the nucleotide code governing how many LIS1 dimers can bind add further complexity. As illustrated in Figure 1, these intermediates may bias dynein toward distinct activation routes, including a classical off-microtubule LIS1-mediated pathway and a microtubule-assisted assembly pathway, but how cells partition between these routes remains unclear. Moreover, it is still unknown how these regulatory inputs are integrated over time during multiple catalytic cycles. Addressing these questions will require a view that treats dynein not as a sequence of discrete states but as an ensemble of allosterically connected conformations, in which nucleotide occupancy, microtubule engagement, load, and LIS1/Nde1 binding dynamically reshape the probabilities of interconverting substates across the entire dimer.

## 4 Toward an ensemble model of dynein mechanochemical cycle

Emerging artificial intelligence (AI-) based techniques and computational methods can leverage experimentally determined structures to enhance our understanding of dynein’s functional motions by mapping out the dynamics and heterogeneity on the conformational free energy landscape. The open questions raised in the preceding section define the target problems that computational approaches must address, including the coupling between linker bending, AAA1 pocket relaxation and stalk registry switching, the basis of interhead communication, and the adaptor-dependent assembly of dynein complexes. However, the available approaches differ substantially in their chemical resolution and accessible timescales, and in the extent to which they provide information on transition kinetics and structural ensembles. Coarse-grained (CG) and structure-based models (SBM) can probe larger collective motions over microsecond-to-millisecond regimes, whereas atomistic MD and enhanced-sampling approaches typically resolve transitions or local free-energy barrier crossings over nanosecond-to-microsecond timescales. At present, no single approach captures the full dynein mechanochemical cycle, which likely unfolds over much slower effective timescales than those directly accessible to most approaches. In early 2010s, computational studies using anisotropic and elastic network models identified collective motions that coupled closure of AAA1-AAA2 cleft to linker repositioning and stalk registry shifts to microtubule binding changes through sliding of coiled coil into localised rearrangements in MTBD helices (Choi et al., 2011; Zheng, 2012; Rao et al., 2019). In parallel, molecular dynamics flexible fitting (MDFF) approach in combination with targeted MD (TMD) simulations was used to generate pseudo atomic models from subnanometer cryo-EM density maps of the MTBD bound to microtubule complex (Redwine et al., 2012). These reconstructions highlighted a large repositioning of MTBD helices and identified dynamic salt bridges switching between intramolecular MTBD contacts (favouring weak binding) and MTBD-tubulin contacts (favouring strong binding).

These early works set the template for subsequent studies using CG-SBM, which enabled simulations of conformational transitions across the full motor domains. Multiple-basin SBMs connecting pre and post powerstroke structures showed that the allosteric cascade propagates clockwise around the AAA+ ring (from linker/AAA1 to AAA6/C-terminal) during the powerstroke, whereas in the recovery stroke it propagates bidirectionally from the AAA6/C-terminal (Kubo et al., 2017). Tirumalai and coworkers used a similar CG approach to uncover a repressed state in which interactions between the linker and insert loops in the AAA2 domain prevent pre powerstroke like state when AAA3 is ATP bound, explaining the gating role of AAA3 (Goldtzvik et al., 2018). CG simulations further showed that AAA3 governs allosteric propagation of conformational changes from AAA1 to MTBD through AAA4/AAA5, and that the linker prefers a straight conformation under these conditions (Dutta and Jana, 2019). A recent hybrid MD/metadynamics/multi-basin SBM study further suggested that, on microtubules, stalk registry shifts can precede MTBD rearrangement during affinity switching (Das and Jana, 2024). Wang *et al.* extended CG-SBM to the dynein dimer, demonstrating that interhead tension coordinates the two motor domains during processive stepping and creates opposite torques on leading and trailing stalks, driving the trailing head from high affinity α registry to low affinity β registry (Wang et al., 2018). Collectively, these studies support the view that dynein contains a topology-driven network capable of coupling linker, ring, stalk, and MTBD motions, and provide mechanistic hypotheses for both intramolecular coupling and inter-head communication. However, SBMs and related Go-like models encode simplified native state energetics and favour predefined endpoint basins, and hence, the inferred sequence of events, barrier heights, and state populations should therefore be viewed as model-dependent rather than definitive. Thus, CG approaches are advantageous in identifying qualitatively plausible pathways and mechanical couplings, but less definitive in establishing causal order, barrier heights or chemically specific energetics.

Jana and coworkers studied ATP hydrolysis in AAA1 using quantum mechanics/molecular mechanics (QM/MM) simulations, which treated the active site components at the density functional or semi-empirical level and the rest of the system with a classical force field (Manna et al., 2020). They identified a conserved glutamate residue (E1742), and two ordered water molecules as key participants in the catalytic mechanism, revealing an associative-like phosphoryl transfer pathway and quantifying the activation barrier for hydrolysis. This study provides atomistic insight into the local chemistry of AAA1, but not into how hydrolysis is coupled to the larger interdomain transitions of the full motor. Recently, MD simulations and enhanced sampling methods (e.g., umbrella sampling, metadynamics) have been used to characterise the large-scale domain transitions at the atomic scale and quantify the associated conformational free energy landscape. Golcuk *et al.* used steered MD and umbrella sampling on human dynein-2 to reconstruct the free energy surface associated with the linker bending and quantified stored elastic energy in the priming stroke. The study revealed multiple bent states separated by a ∼5.7 K_B_T barrier and showed that the AAA+ ring can impose a steric constraint that prevents spontaneous reversal once the linker is primed (Golcuk et al., 2024).

All-atom MD simulations have also been applied to understand how regulatory proteins reshape dynein’s conformational energy landscape, particularly in the context of LIS1-dependent activation. MD simulations indicate that the cancer-correlated D338G substitution in LIS1 and K2879A mutation in human dynein reduce binding energy between dynein and LIS1 (Ton et al., 2023). Further, these data indicate that the conformational changes in the ring that result from microtubule binding likely account for disruption of the dynein-LIS1 complex. In a complementary approach, Leschziner and coworkers combined deep learning method cryoDRGN with Gaussian accelerated MD (GaMD) to identify and resolve a conformational landscape of bent, intermediate and straight linker states together with their AAA+ nucleotide occupancies (Kendrick et al., 2025). CryoDRGN uses variational autoencoders (VAEs) for modelling complex ensembles containing both continuous and discrete heterogeneity and reconstructs continuous distributions of 3D density maps from single-particle cryo-EM datasets (Zhong et al., 2021). The cryo-EM derived classes then served as the starting structures for GaMD simulations, thereby providing an experimentally anchored route for linking structural heterogeneity with computed free-energy landscapes. Using this approach, the authors demonstrated how LIS1 constrains linker rotation and loosens the AAA3-AAA4 interface, facilitating ADP release from AAA3 and accelerating dynein’s basal ATPase rate. More broadly, this approach addresses the regulator-dependent questions raised earlier, as it can compare experimentally observed substates and ask how an adaptor reweights the conformational ensemble. Nevertheless, cryo-EM heterogeneity analysis reports structural distributions rather than transition kinetics, and the associated MD refinement remains limited to the local transitions and state connectivity that can be sampled from those experimentally derived starting points.

## 5 Discussion

Over the past decade, structural, biophysical, and computational studies have transformed cytoplasmic dynein-1 from a poorly understood minus-end motor into well characterised mechanoenzyme in the cell. High resolution X-ray and cryo-EM structures, together with coarse-grained and atomistic simulations have revealed an allosteric network linking different subunits of dynein to functional and non-functional states stabilised by cofactors such as dynactin, cargo adaptors, LIS1, and Nde1 (Okada et al., 2023; Yang et al., 2026). Taken together, these studies describe dynein function across multiple resolutions, from low-resolution structural models to quantum mechanical and atomistic descriptions, and suggest that linker bending, AAA1 pocket closure, stalk registry shifts, and MTBD affinity changes are only partially coupled, while interhead coordination in dimers emerges from probabilistic load sharing rather than a fixed stepping pattern (Belyy et al., 2014). Recent progress in experimental techniques, GPU-accelerated computing, structure prediction tools and deep generative models to sample ensemble distributions makes it plausible to map conformational diversity and bridge experiment with multiscale dynamics (Baek et al., 2021; Shugaeva et al., 2025; Jumper et al., 2021; Jones et al., 2022; Lewis et al., 2025). However, several of these approaches do not resolve how to quantitatively weigh thermodynamics and kinetics of alternative substates, nor integrate information from experiments that probe different time and length scales. Hence, the mechanistic gaps inferred from any single method must be treated as hypotheses rather than an established mechanism.

Integrative modelling using Bayesian inference methods has emerged as a strategy to bridge this gap by combining prior physicochemical knowledge with heterogeneous experimental data such as cryo-EM, SAXS, NMR, etc (Bonomi et al., 2016; Gusew et al., 2026). Here, MD simulations are restrained against experimental observables (e.g., cryo-EM density maps), yielding weighted structural ensembles without overfitting noisy or flexible regions (Bonomi and Vendruscolo, 2019). These frameworks have been extended with enhanced sampling techniques to overcome high free energy barrier in complex macromolecular systems (Brotzakis et al., 2023) or with the use of AlphaFold predicted inter-residue distances as restraints, generating conformational ensembles for intrinsically disordered proteins (Brotzakis et al., 2025). Such an approach, if applied to dynein, could connect the resulting local ensembles across the bent, intermediate and straight cryo-EM classes. It would provide a thermodynamically weighted description of the allosteric cascade and insights into regulator-specific effects and disease-linked mutations. Pathogenic substitutions in dynein, dynactin, or LIS1 would then be interpreted as local perturbations that can reweight allosteric pathways, or modulate existing conformational free energy landscape, thus providing a mechanistic basis for linking structural alterations to phenotypes.
